# Whole Irradiated Plant Leaves Showed Faster Photosynthetic Induction Than Individually Irradiated Leaves *via* Improved Stomatal Opening

**DOI:** 10.3389/fpls.2019.01512

**Published:** 2019-11-28

**Authors:** Shunji Shimadzu, Mitsunori Seo, Ichiro Terashima, Wataru Yamori

**Affiliations:** ^1^Department of Biological Sciences, Graduate School of Science, The University of Tokyo, Tokyo, Japan; ^2^RIKEN Center for Sustainable Resource Science, Yokohama, Japan; ^3^Institute for Sustainable Agro-Ecosystem Services, The University of Tokyo, Nishitokyo, Japan

**Keywords:** photosynthesis, photosynthetic induction, stomatal conductance, systemic signaling, abscisic acid

## Abstract

Rapid photosynthetic induction is crucial for plants under fluctuating light conditions in a crop canopy as well as in an understory. Most previous studies have focused on photosynthetic induction responses in a single leaf, whereas the systemic responses of the whole plant have not been considered. In a natural environment, however, both single leaves and whole plants are exposed to sunlight, since the light environment is not uniform even within a given plant. In the present study, we examined whether there is any difference between the photosynthetic induction response of a leaf of a whole irradiated plant and an individually irradiated leaf in *Arabidopsis thaliana* to consider photosynthetic induction as the response of a whole plant. We used two methods, the visualization of photosynthesis and direct measurements of gas-exchange and Chl fluorescence, to demonstrate that whole irradiated plant promoted its photosynthetic induction *via* improved stomatal opening compared with individually irradiated leaf. Furthermore, using two *Arabidopsis* knockout mutants of abscisic acid transporter, *abcg*25 and *abcg*40, the present study suggests that abscisic acid could be involved in this systemic response for stomatal opening, allowing plants to optimize the use of light energy at minimal cost in plants in a dynamic light environment.

## Introduction

Plant biomass is determined by the total incident radiation that occurs during the growing season, the light-interception efficiency of a plant, and the conversion efficiency of the intercepted radiation into biomass (Long et al., 2006; Zhu et al., 2010). The last factor, namely the conversion efficiency, is considered to be primarily determined by photosynthesis. As the light condition in a natural environment changes dynamically over time, the leaf photosynthetic rate does not always reach its steady state. Photosynthetic reactions, including stomatal opening and the enzymatic reaction, are switched off in the dark, specifically to prevent (1) water loss from stomata and (2) the unnecessary metabolism of carbon assimilation. Thus, plant leaves need some time to open their stomata and reactivate the enzymes of carbon assimilation when the irradiance increases rapidly in light-flecked environment after a prolonged period of darkness. The photosynthetic rate increases gradually over several minutes and approaches a new steady state when the light intensity on a leaf is increased suddenly after a prolonged period of low light or darkness. This phenomenon has been termed “photosynthetic induction” (Pearcy, 1990), which occurs both in crop canopies and forest understories (Schurr et al., 2006).

The photosynthetic induction response can typically be divided into three phases that are highly interactive with each other: (1) photosynthetic electron transport, which is usually complete within the first 1–2 min, (2) enzyme reactions in a Calvin–Benson cycle, which often takes 5–10 min, and (3) stomatal opening, which typically takes as much as 1 h (Pearcy, 1990; Yamori, 2016). Recent studies have shown that cyclic electron flows around photosystem I (Yamori et al., 2016c) as well as ion channels such as KEA3, a thylakoid membrane localized K^+^/H^+^ antiporter (Armbruster et al., 2014; Kunz et al., 2014), and VCCN1, a voltage-dependent Cl^−^ channel (Herdean et al., 2016) are involved in photosynthetic induction to adjust photosynthetic light utilization in electron transport under fluctuating light conditions (for a review, see Tanaka et al., 2019). Moreover, it has been shown that Rubisco activase, an enzyme involved in Rubisco activation, is essential for photosynthetic induction in the second phase (Mott and Woodrow, 2000; Yamori et al., 2012; Carmo-Silva and Salvucci, 2013; Kaiser et al., 2016). In addition, the stomatal opening can be another factor limiting photosynthetic induction, as stomatal responses are much slower than the activation process of a Calvin cycle (Allen and Pearcy, 2000a; Allen and Pearcy, 2000b; Lawson et al., 2012; Kaiser et al., 2016).

The conversion efficiency of intercepted radiation into biomass under fluctuating light conditions is important for plant growth, especially for crops and for the survival of understory plants (Tinoco-Ojanguren and Pearcy, 1993; Valladares and Pearcy, 1997; Urban et al., 2007; Montgomery and Givnish, 2008). In particular, rapid photosynthetic induction improves the energy gain for CO_2_ assimilation in dark‐adapted leaves exposed to light flecks, since light flecks contribute up to 60–80% of the photosynthetically active radiation experienced by understory plants (Pearcy and Seemann, 1990; Leakey et al., 2003; Leakey et al., 2005). Additionally, the enhancement of photosynthetic capacity under fluctuating light has been receiving much attention, as an understanding of the physiological and genetic mechanisms behind photosynthetic induction is expected to contribute to it (Tanaka et al., 2019). Most previous studies have focused on photosynthetic induction responses in a single leaf, and the systemic responses of the whole plant have not been considered. In a natural environment, however, both single leaves and whole plants are exposed to sunlight, and light environments are not uniform even within a plant. In fact, different plant parts can communicate with one another through specific signals, which is known as systemic signaling (Karpinski et al., 1999; Białasek et al., 2017). Previous studies have shown that the uppermost leaves, which are generally the first to receive sunlight, display faster photosynthetic induction than understory leaves (Bai et al., 2008). Photosynthetic induction in understory leaves is enhanced by the preillumination of upper leaves but not lower leaves (Hou et al., 2015). Furthermore, preillumination of a shoot apex could accelerate photosynthetic induction in distal leaves (Guo et al., 2016). These researches implied that the photosynthetic response to fluctuating light in a single leaf would be different from that in the leaves of a whole plant.

In this study, we examined whether there is any difference between the photosynthetic induction responses of the leaf of a plant where all the leaves were irradiated (WIP, whole irradiated plant), and a leaf of a plant where all the other leaves were kept in the dark (IIL, individually irradiated leaf), in *Arabidopsis thaliana* to consider photosynthetic induction as a response of the whole plant. We also focused on abscisic acid (ABA) transport as a possible of systemic signaling mechanism in photosynthetic induction, since ABA is known to play pivotal roles in the regulation of stomatal opening/closing. Using two *Arabidopsis* knockout mutants, *abcg*25, which is an ABA exporter mediating the ABA efflux from vascular tissues (Kuromori et al., 2010) and *abcg*40, which is an ABA importer expressed in guard cells (Kang et al., 2010), we analyzed the relationship between ABA and photosynthetic induction and the effect of ABA on systemic signaling. These studies will provide a new perspective for a strategy that will enable plants to improve the light utilization efficiency of photosynthesis in crop canopies and forest understories.

## Material and Methods

### Plant Materials and Growth Conditions

The *A. thaliana* mutants, *abcg25* (SALK_063716) (Kanno et al., 2012), *abcg40-2* (SALK_005635) (Kang et al., 2010), *aba3-1* (Lléon1996on-Kloosterziel, et al., 1996), and the wild type (Col-0), were grown in soil in an environmentally controlled growth chamber. ABCG25 is localized in a plasma membrane in vascular tissue and executes ABA transport from the vasculature, ABCG40 is localized in guard cells and functions as a plasma membrane ABA uptake transporter, and ABA3-1 is impaired ABA synthesis. All the plants were grown in a 200-ml plastic pot containing soil, and each pot was supplied once a week with 100 ml of 1/500 strength nutrient solution (HYPONeX, N/P/K, 6:10:5, Hyponex Japan, Osaka, Japan). The growth chamber was operated at an air temperature of 23°C, a relative humidity of 70%, a photosynthetically active photon flux density (PPFD) of 150 µmol m^−2^ s^−1^, with an 8-h photoperiod and a CO_2_ concentration of 400 µmol mol^−1^. The rice (*Oryza sativa* cv. Hitomebore) was grown in a 1.3-L plastic pot with 1.0 g of slow-release fertilizer (Temairazu; Co-op Chemical Co., Ltd., Tokyo, Japan). The growth chamber was also environmentally controlled and operated at a temperature of 23°C, a relative humidity of 70%, a PPFD of 500 µmol m^−2^ s^−1^, with a 14-h photoperiod and a CO_2_ concentration of 400 µmol mol^−1^. All plants were given enough water, however, ABA-deficient mutant, *aba3-1*, was susceptible to water stress as shown previously (Finkelstein et al., 2002).

### Analysis of Gas Exchange and Chlorophyll Fluorescence

Gas exchange and chlorophyll fluorescence were concurrently measured at a cuvette temperature of 25°C and a relative humidity of 70%, in fully expanded young leaves with a portable gas exchange system LI-6400XT (Li-COR, Lincoln, NE, USA). A single leaf was clamped by the chamber of the Li-6400XT and the photosynthetic parameters were measured. First, leaves of the plants that were maintained in darkness overnight were treated with a saturating pulse to obtain maximum fluorescence. Then, the quantum yield of photosystem II [Y(II)], which reflects the photochemical efficiency of the electron transfer through photosystem II and the fraction of the oxidized photosystem II centers (qP), were obtained at 500 µmol m^−2^ s^−1^ for *A. thaliana* or 1,000 µmol m^−2^ s^−1^ for rice, as described previously (Baker, 2008). The electron transport rates (ETRs) through photosystem II were calculated using the following equation: ETR = 0.5 × abs I × Y(II), where 0.5 is the fraction of absorbed light allocated to photosystems, and abs I refers to the absorbed irradiance taken as 0.84 of incident irradiance.

### A–C_i_ Curve

*A–C*_i_ curve (CO_2_ assimilation rate, *A*, versus intercellular CO_2_ concentrations, *C* *_i_*) analysis was performed at 500 µmol m^−2^ s^−1^ with an LI-6400XT. First, the steady-state photosynthetic rate at a CO_2_ concentration of 400 μmol mol^−1^ was measured, and the CO_2_ concentration was changed successively to 100, 200, 300, 400, 600, 800, 1,200, and 1,500 μmol mol^−1^. The photosynthetic rates were recorded after 5 min exposure to each CO_2_ concentration.

### Imaging PAM

Chlorophyll fluorescence was measured with an imaging fluorometer (IMAGING-PAM; Heinz Walz) in 4 to 6-week old plants. The plants were kept in darkness overnight, and then the photosynthetic induction response at a PPFD of 1,000 µmol m^−2^ s^−1^ was measured. The quantum yield of photosystem II [Y(II)], nonphotochemical quenching, and the fraction of oxidized photosystem II centers (qP) were analyzed.

### Light Conditions for the Measurement of Photosynthetic Induction

**Figure 1:**
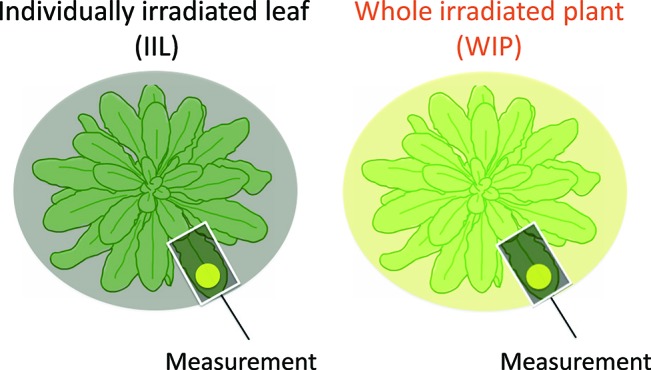
Schematic diagram of light treatment to a leaf of a whole irradiated plant (WIP) and an individually irradiated leaf (IIL). During the measurement of the IIL, the other leaves were covered with black cloth and were kept in the dark, whereas during the measurement of the WIP, all the leaves of a plant were irradiated. Under both light conditions, the selected target leaves were similar in age.

We recorded the photosynthetic induction response in a WIP, in which all the leaves were irradiated, and in an IIL, where all the other leaves were kept in the dark. The rate of photosynthetic induction of an IIL and a WIP was compared. For the light treatment of the individual leaf, the IIL was clamped in a cuvette of the Li-6400XT while the rest of the plant remained in darkness. In contrast, for the light treatment of the whole plant, a leaf of the WIP was clamped while the rest of the plant was illuminated with the same light intensity by the same light source during the photosynthetic induction measurement (**Figure 1**). By using these plants, which had been kept in the dark overnight, the responses of various photosynthetic parameters to an irradiance of 500 µmol m^−2^ s^−1^ for *A. thaliana* or 1,000 µmol m^−2^ s^−1^ for rice were measured every 30 s.

### Plant Growth Analysis

The plants were grown in a growth chamber at room temperature (23°C), a relative humidity of 70%, a PPFD of 150 µmol m^−2^ s^−1^ and an 8-h photoperiod until 23 days after sowing. Then, the plants were transferred to either fluctuating light conditions or constant light conditions. Under both conditions, the plants were exposed to a high light intensity of 500 μmol m^−2^ s^−1^ for 4 h and a low light intensity of 60 μmol m^−2^ s^−1^ for 8 h per day. Under the constant light conditions, the plants were exposed to a low light intensity for 4 h each in the morning and evening, and to a high light intensity for 4 h around midday. On the other hand, under the fluctuating light conditions, a high light intensity for 5 min and a low light intensity for 10 min were alternated for 12 h. At 43 days after sowing, the above-ground parts of the plants were sampled and dried at 80°C for several days, and their dry weights were measured.

## Results

### Photosynthetic Induction in IIL and WIP

**Figure 2:**
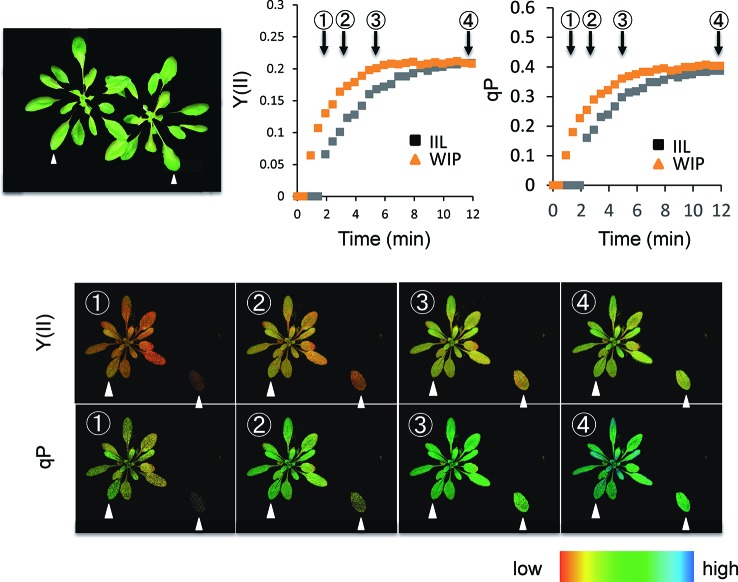
Time-course imaging of photosynthetic induction by Imaging-PAM in WT of *Arabidopsis thaliana*. The photosynthetic induction response at an intensity of 1,000 µmol m^−2^ s^−1^ was measured after keeping the sample in darkness overnight. During the analysis of Imaging-PAM, for the IIL measurement, leaves other than the target leaf were covered with a black cloth and kept in the dark, whereas for the WIP measurement all the leaves of a plant were irradiated. The quantum yield of photosystems II [Y(II)] and the fraction of reduced photosystem II centers (qP) were recorded every 20 s. The colored bar indicates the value range.

Photosynthetic induction was compared between an IIL and a leaf of WIP (**Figure 1**). During the analysis with Imaging-PAM of *A. thaliana*, for the IIL measurement, the other leaves were covered with black cloth and were kept in the dark (**Figure 2**), whereas, for the WIP measurement, whole plant was irradiated during the measurement of photosynthetic induction of the targeted leaf, which was a similar age to the IIL. The WIP significantly promoted the induction of Y(II) and qP upon exposure to a high light intensity (1,000 μmol m^−2^ s^−1^) at a CO_2_ concentration of 400 μmol mol^−1^ (**Figure 2**), indicating that the WIP would be able to use more light energy to drive the electron transport to generate adenosine triphosphate and reduced nicotinamide adenine dinucleotide phosphate during the first few minutes after a change in light intensity.

**Figure 3:**
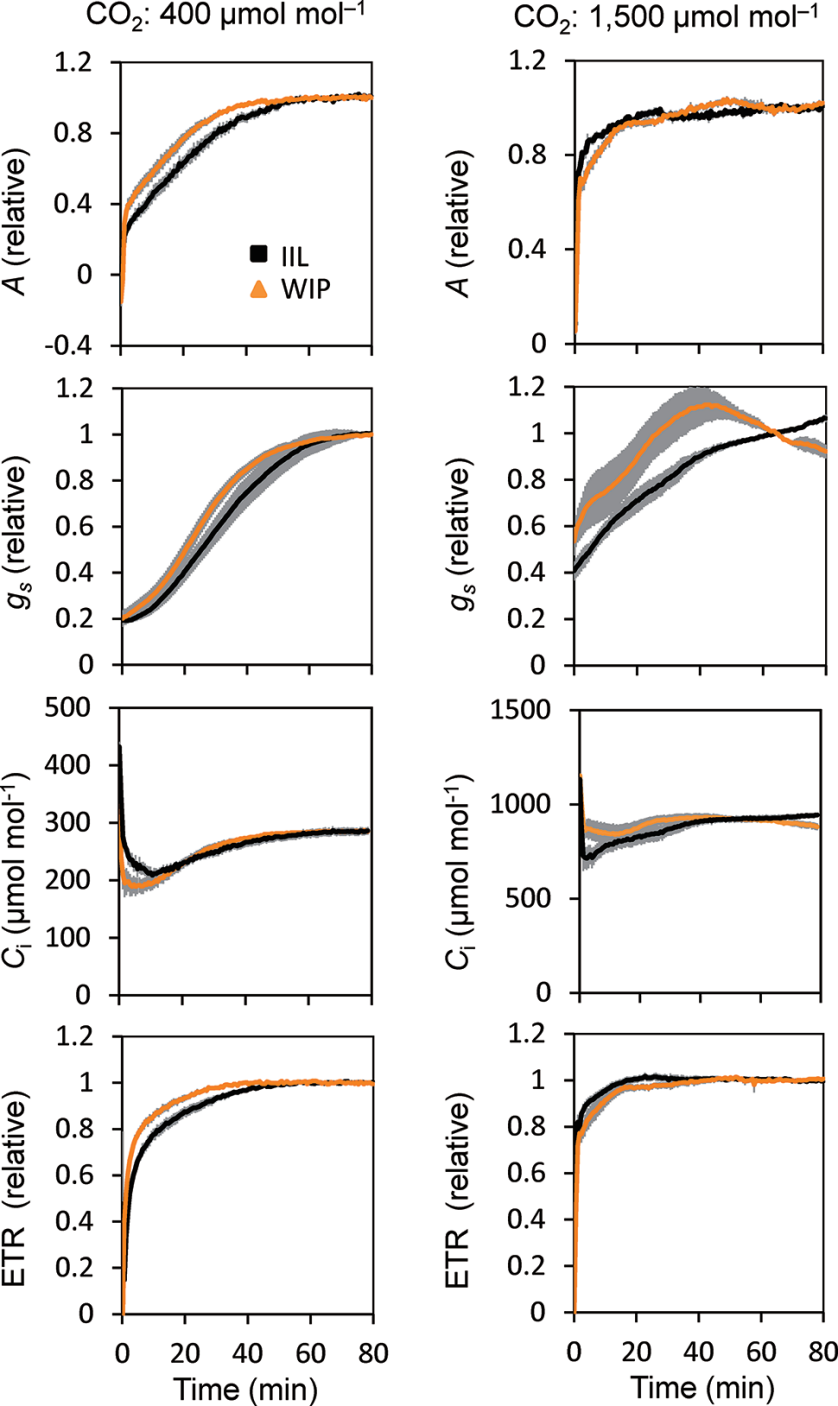
Photosynthetic induction of IIL and WIP of *Arabidopsis* WT. CO_2_ assimilation rate (*A*), stomatal conductance (*g*_s_), intercellular CO_2_ concentration (*C*_i_), and photosynthetic electron transport rate (ETR) were simultaneously measured in an IIL or WIP, at CO_2_ concentrations of 400 μmol mol^–1^ and 1,500 μmol mol^–1^. The leaves of plants kept in the dark overnight were used for the experiments. The photosynthetic parameters were recorded every 30 s at an irradiance of 500 μmol photons m^–2^ s^–1^ for a total of 80 min. Absolute values are shown in **Figure S1**. The data are the means ± standard errors of four biological replicates.

This was supported by the concomitant measurement of gas exchange and Chl fluorescence, which showed that the WIP exhibited a faster induction of CO_2_ assimilation (*A*) and photosynthetic ETR at a high light intensity of 500 μmol m^−2^ s^−1^ at a CO_2_ concentration of 400 μmol mol^−1^ in *Arabidopsis* (**Figure 3**). Moreover, the steady-state *A* and ETR tended to be greater in the WIP than in the IIL (**Figure S1**). Interestingly, the WIP significantly promoted the induction of stomatal conductance (g_s_) upon exposure to a high light intensity and thus the transition to the steady-state of the intercellular CO_2_ concentrations (*C* *_i_*) was faster in the WIP than in the IIL. The WIP significantly shortened the time required to reach 60% (T_60_) of the maximum *A*, *g* *_s_*, and *ETR*, and the time required to reach 60% in the transition from minimum to maximum *C* *_i_* (**Table 1**). This result was also confirmed in rice (**Figure S2**). In contrast, the WIP lost its effect upon exposure to a high light intensity at a high CO_2_ concentration of 1,500 μmol mol^−1^, where the effect of the stomatal response on photosynthetic induction could be negligible since *C* *_i_* was held above a certain level regardless of the stomatal response (**Figure 3**). These findings indicate that the WIP promoted its photosynthetic induction *via* an improvement in the stomatal response.

**Table 1:** The time required to reach 60% (T_60_) of the maximum CO_2_ assimilation rate (*A*), stomatal conductance (*g* *_s_*), the intercellular CO_2_ concentrations (*C* *_i_*), and photosynthetic electron transport rate (ETR) at a CO_2_ concentration of 400 or 1,500 μmol mol^−^^1^ between in a leaf of a whole irradiated plant (WIP) and an individually irradiated leaf (IIL) in WT of *Arabidopsis thaliana*.

T_60_ (min)	IIL	WIP
*A* *_400_*	18.3 ± 2.5	10.8 ± 1.8*
*g* *_s400_*	37.0 ± 2.97	29.0 ± 1.73*
*C* *_i400_*	32.5 ± 2.36	24.7 ± 1.25*
*ETR* *_400_*	3.53 ± 0.34	1.72 ± 0.20*
*A* *_1500_*	0.95 ± 0.12	1.16 ± 0.10
*ETR* *_1500_*	0.79 ± 0.08	0.92 ± 0.03

The values are presented as the mean ± standard deviation (n ≥ 4), and the asterisks next to WIP indicate significant differences between data for the IIL and WIP (Student’s t-test, P < 0.05).

### Photosynthetic Induction in ABCG Knockout Mutants

**Figure 4:**
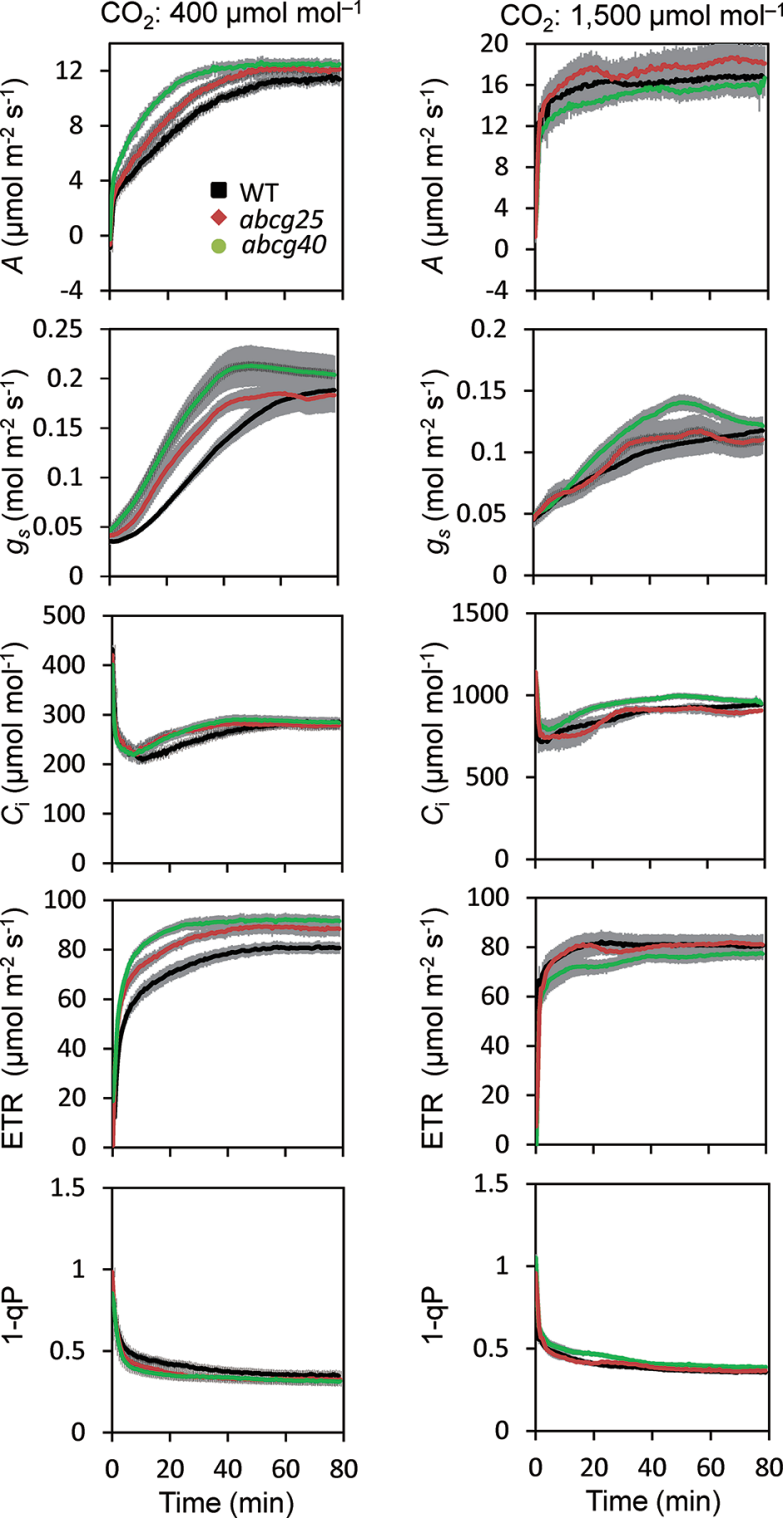
Photosynthetic induction in IIL among WT and two *abcg* mutants. CO_2_ assimilation rate (*A*), stomatal conductance (*g*_s_), intercellular CO_2_ concentration (*C*_i_), photosynthetic electron transport rate (ETR), the redox state of the plastoquinone pool (1 − qP) were simultaneously measured at CO_2_ concentrations of 400 and 1,500 μmol mol^−1^. The data are the means ± standard errors of four biological replicates.

During the photosynthetic induction, the stomata opened synchronously as the CO_2_ assimilation accelerated. To clarify the role of the stomata in the promotion of photosynthetic induction, and to evaluate whether ABA plays pivotal roles in the promotion of photosynthetic induction, we compared the photosynthetic induction processes at a CO_2_ concentration of 400 μmol mol^−1^ of wild type (WT) and two *Arabidopsis* knockout mutants of ABA transporter, *abcg*25 and *abcg*40. Photosynthetic CO_2_ response curves (*A - Ci* curve) were similar among WT and two *abcg* mutants (**Figure S3**).The rate at which *A* and *g* *_s_* approached a steady state following an increase in the irradiance was fastest in *abcg40*, intermediate in *abcg25*, and slowest in WT (**Figure 4**). This was supported by the time required to reach 60% (T_60_) of the maximum *A*, *g* *_s_*, and ETR, and the time required to reach 60% of the maximum *C* *_i_* compared to the minimum upon irradiation at a CO_2_ concentration of 400 μmol mol^–1^ (**Table 2**). The rates at which the reduction level of the plastoquinone pool (1 − qP) approached their steady states upon irradiation were faster in the two *abcg* mutants than in WT. These results indicate that, during photosynthetic induction, the two mutants utilized more light energy driving photosynthesis.

**Table 2:** The time required to reach 60% (T_60_) of the maximum CO_2_ assimilation rates (*A*), stomatal conductance (*g* *_s_*), the intercellular CO_2_ concentrations (*C* *_i_*), and photosynthetic electron transport rate (ETR) at a CO_2_ concentration of 400 or 1,500 μmol mol^−^^1^ in an individually irradiated leaf (IIL) in WT, *abcg25*, and *abcg40* knockout mutants.

T_60_ (min)	WT	*abcg 25*	*abcg 40*
*A* *_400_*	18.3 ± 2.5 a	14.3 ± 2.6 ab	7.7 ± 1.4 b
*g* *_s400_*	37.0 ± 2.97 a	27.6 ± 2.35 ab	24.9 ± 3.42 b
*C* *_i400_*	32.5 ± 2.36 a	17.9 ± 0.79 b	23.6 ± 2.40 b
*ETR* *_400_*	3.53 ± 0.34 a	2.30 ± 0.47 b	2.36 ± 0.32 ab
*A* *_1500_*	0.95 ± 0.12 a	1.27 ± 0.09 a	1.19 ± 0.10 a
*ETR* *_1500_*	0.79 ± 0.08 a	1.06 ± 0.08 a	1.03 ± 0.04 a

The values are presented as the mean ± standard deviation (n ≥ 4), and the different letters denote significant differences (Tukey–Kramer’s honest significant difference test).

We also compared the photosynthetic induction process for WT, *abcg25* and *abcg40* at a high CO_2_ concentration of 1,500 μmol mol^−1^ (**Figure 4**). The induction response of all the photosynthetic parameters (i.e., *A*, *g* *_s_*, and ETR) showed no clear difference for WT and the two *abcg* mutants, which was supported by the T_60_ of the maximum *A*, *g* *_s_*, and *C* *_i_* at a CO_2_ concentration of 1,500 μmol mol^−1^ (**Table 2**). These results showed that stomatal opening would actually have a great influence on the photosynthetic induction process. This was partly supported by a previous study, which reported that increases in initial *g* *_s_* up to a threshold value accelerate photosynthetic induction in a knockout mutant of ABA synthesis, *aba*2-1 (Kaiser et al., 2016).

**Figure 5:**
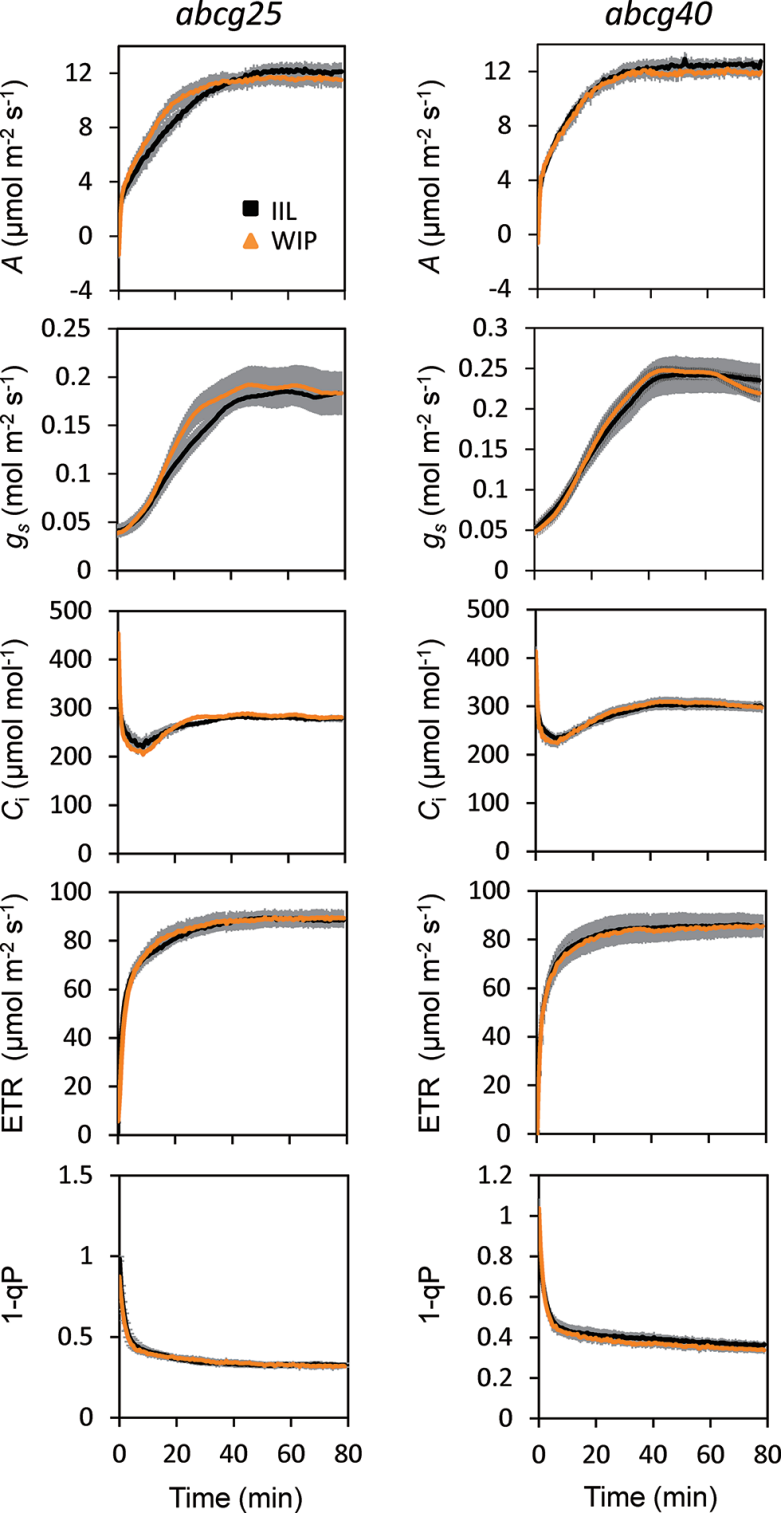
Photosynthetic induction of IIL and WIP in two *abcg* mutants. CO_2_ assimilation rate (*A*), stomatal conductance (*g*_s_), intercellular CO_2_ concentration (*C*_i_), photosynthetic electron transport rate (ETR), the redox state of the plastoquinone pool (1 − qP) were simultaneously measured in an IIL or WIP, at a CO_2_ concentration of 400 μmol mol^–1^. The leaves of plants kept in the dark overnight were used for experiments. The photosynthetic parameters were recorded every 30 s at an irradiance of 500 μmol photons m^–2^ s^–1^ until 80 min. The data are the means ± standard errors of four biological replicates.

To further examine whether ABA is involved in the stomatal responses observed in the WIP and IIL, we compared the photosynthetic induction processes of *abcg25* and *abcg40* knockout mutants under two conditions at a CO_2_ concentration of 400 μmol mol^–1^. In the two *abcg* mutants, the induction responses of all the photosynthetic parameters (i.e., *A*, *g* *_s_*, and ETR) were similar for the WIP and IIL (**Figure 5**, **Table 3**), although it was significantly different in WT (**Figure 3**). Taken together, these results suggest that the promotion of photosynthetic induction by whole plant irradiation is affected by stomatal responses to ABA, which are regulated by the two ABA transporters.

**Table 3:** The time required to reach 60% (T_60_) of the maximum CO_2_ assimilation rates (*A*), stomatal conductance (*g* *_s_*), the intercellular CO_2_ concentrations (*C* *_i_*), and photosynthetic electron transport rate (ETR) at a CO_2_ concentration of 400 μmol mol^−^^1^ in a leaf of a whole irradiated plant (WIP) and an individually irradiated leaf (IIL), and in both *abcg25* and *abcg40* knockout mutants.

T_60_ (min)		IIL	WIP
*abcg25*	*A* *_400_*	14.3 ± 2.6	9.1 ± 1.4
	*g* *_s400_*	27.6 ± 2.35	21.8 ± 1.57
	*C* *_i400_*	17.9 ± 0.79	18.1 ± 2.25
	*ETR* *_400_*	2.30 ± 0.47	2.76 ± 0.21
*abcg40*	*A* *_400_*	7.70 ± 1.36	8.66 ± 1.68
	*g* *_s400_*	24.9 ± 3.41	23.1 ± 1.83
	*C* *_i400_*	23.6 ± 2.40	20.3 ± 1.93
	*ETR* *_400_*	2.36 ± 0.32	2.39 ± 0.31

The values are presented as the mean ± standard deviation (n ≥ 4). There was no statistical difference between theT_60_ values for the IIL and WIP and in both abcg25 and abcg40 (Student’s t-test, P < 0.05).

### Plant Growth Under Fluctuating Light Conditions in abcg Knockout Mutants

**Figure 6:**
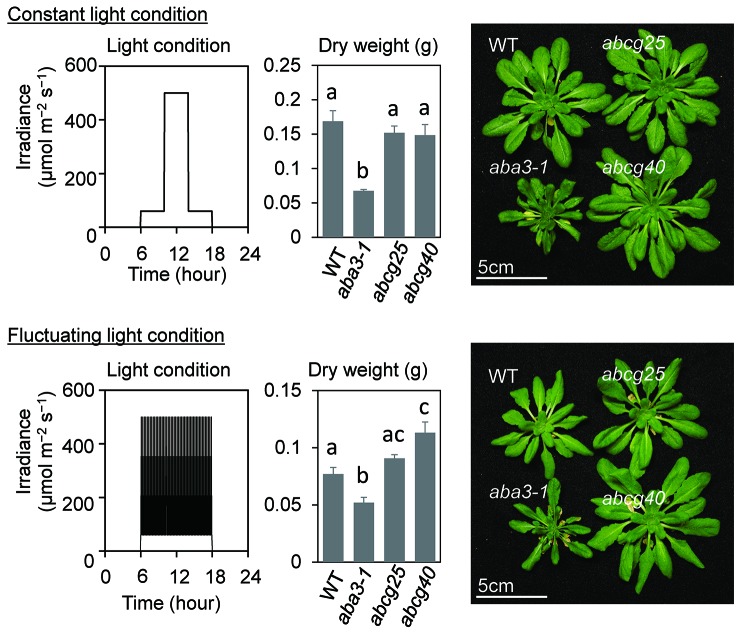
Plant growth under constant light and fluctuating light conditions. The growth light conditions and dry weights of aerial parts of plants at 43 days after sowing (DAS) were shown with plant pictures at 43 DAS. All plants were grown under constant light until 23 DAS and were divided into two growth conditions; constant light and fluctuating light conditions. Under the constant light conditions, the plants were exposed to low light for 4 h in the morning and 4 h in the evening, and to high light for 4 h in the middle of the day, whereas, under the fluctuating light conditions, a high light for 5 min and low light for 10 min were alternated for 12 h. The data are the means ± standard errors of four biological replicates, and the letters denote significant differences (Tukey–Kramer’s honest significant difference test).

To examine the effect of the improvement of the photosynthetic induction response on the total biomass in *abcg* knockout mutants, these mutants as well as wt and *aba3-1*, which impaired aba synthesis, were grown under both fluctuating and constant light conditions. wt and two *abcg* mutants grew almost equally under constant light conditions, whereas the plant growth of the two *abcg* knockout mutants was greater than that of wt under fluctuating light conditions where there was an alternating high light intensity of 500 µmol m^−2^ s^−1^ for 5 min and a low light intensity of 60 µmol m^−2^ s^−1^ for 10 min (**Figure 6**). growth of *aba3-1* knockout mutant was apparently suppressed both in fluctuating light conditions and constant light conditions, since drought stress could suppress its plant growth.

## Discussion

As light flecks are the primary energy source for plants not only in the understory but also in the crop canopy (Pearcy and Seemann, 1990; Pearcy and Way, 2012), rapid photosynthetic induction is crucial for plants under fluctuating light conditions. Light flecks move continuously from one leaf of a plant to another, since light flecks are usually too small to cover a whole plant in a forest understory or canopy. To date, most previous studies have focused on the photosynthetic induction responses in a single leaf, with scant attention to the systemic responses of the whole plant. Here, we used two methods, the visualization of photosynthesis with Imaging-PAM and direct measurements of gas exchange and Chl fluorescence with an Li-6400XT, to demonstrate that a leaf of a WIP promotes photosynthetic induction *via* improvement of the stomatal response in comparison with an IIL. This mechanism is important for optimizing the light utilization efficiency of photosynthesis at minimum cost in plants in a dynamic light environment. In addition, a better understanding of the photosynthetic induction response is necessary if we are to better calculate the terrestrial carbon cycle and its influence on the atmospheric CO_2_ concentration and global climate change, since most photosynthetic models used for global carbon issues are based on steady-state photosynthesis under constant environmental conditions in single leaves.

### Whole Irradiated Plants Exhibited Faster Photosynthetic Induction *via* Improved Stomatal Opening

This study showed that the photosynthetic induction time at 400 μmol mol^–1^ CO_2_ was shortened with a rapid increase in *g*_s_ in a WIP compared with an IIL, whereas at a high CO_2_ concentration of 1,500 μmol mol^−1^, the photosynthetic induction time was shortened under both conditions and the differences in photosynthetic induction times were eliminated (**Figure 3**). These results clearly showed that the reduction in the photosynthetic induction time in a WIP was caused by the quick stomatal opening.

It has been suggested that ABA is actively synthesized in leaf vascular tissues and then transported to guard cells to close the stomata in response to water stress, although guard cell autonomous ABA biosynthesis has also been reported (Kuromori et al., 2018). *Arabidopsis ABCG25*, which encodes an ABA exporter, is expressed in vascular tissues (phloem companion cells) (Kuromori et al., 2010; Kuromori et al., 2014) whereas *ABCG40*, which encodes an ABA importer, is expressed in guard cells (Kang et al., 2010). Thus, it is expected that both *abcg25* and *abcg40* would have lower ABA concentrations in guard cells. In these mutants, photosynthetic induction time and the increase in *g*_s_ at 400 μmol mol^–1^ CO_2_ were almost the same for the IIL and WIP (**Figure 5**, **Table 3**), and also were much faster than for WT (**Tables 1** and **3**), suggesting that the reduction in the ABA levels within guard cells is involved in the stomatal opening in response to irradiation and that this process is enhanced in a WIP. It has been reported that changes in *g* *_s_* induced by guard cells are linked with ABA signaling arriving in the xylem (Tardieu et al., 1992), and that there are negative correlations between the ABA concentrations in xylem sap and *g* *_s_* (Tardieu et al., 1991). Although most studies have focused on ABA production in roots followed by its transport to leaves *via* transpiration (Tardieu et al., 1992), it is now recognized that ABA is also produced by local biosynthesis in leaves (Boursiac et al., 2013; Takahashi et al., 2018). The importance of ABA as a systemic signal initiating stomatal closure has also been shown (Christmann et al., 2007). Thus, the distribution of ABA in a xylem flow as well as the ABA uptake into guard cells could affect photosynthetic induction, although it is unknown how ABA transport mediated by ABCG25 and ABCG40 is regulated in response to light irradiation. In leaves, stomata typically close at night to limit transpiration and save water, and the stomatal response to darkness might be related to the ABA concentration in guard cells. On the assumption that a low concentration of ABA is present in xylem sap, which could close stomata at night, and that transpiration is promoted upon irradiation only in one leaf of a plant where all the other leaves are kept in the dark (IIL), ABA could be concentrated only in the leaf *via* transpiration, leading to stomatal closure. On the other hand, assuming that transpiration is promoted upon irradiation of all the leaves of a plant (WIP), ABA could not be concentrated, leading to prompt stomatal opening.

In addition to ABA, several other systemic signals such as chemical signals, electrical long-distance signals, and hydraulic signals have been reported (for a review, see Huber and Bauerle, 2016) Recently, it was reported that the induction of photosynthesis and stomatal opening in understory leaves is enhanced by the preirradiation of upper leaves but not lower leaves, suggesting a directional signal transfer passing through the phloem (Hou et al., 2015). Another recent report showed that systemic signaling mediated by phytochrome B and auxin caused by the irradiation of the shoot apex promoted photosynthetic induction (Guo et al., 2016), as the phytohormone auxin is produced in the shoot apex and redistributed throughout the shoot by rapid phloem transport (Ljung et al., 2001) and changes in the light environment can greatly alter auxin homeostasis (Halliday et al., 2009). This systemic signaling might also be related to the differences in the photosynthetic induction of WIP and IIL observed in the present study.

Also, we cannot exclude the possibility that changes in the turgor pressure in mesophyll cells could affect stomatal opening more in a WIP than in an IIL. In general, the more stomata open, the more plants lose water by transpiration. Since a WIP promotes photosynthetic induction and stomatal opening in the entire plant, the plant would lose more water than an IIL where only a single leaf is irradiated. As the water flow in vessels would be a factor limiting the water supply to a leaf, it can be expected that the leaf water content during photosynthetic induction would be lower in a WIP than an IIL. Stomatal opening and closing takes place due to changes in the turgor pressure in guard cells. Solutes are taken in the guard cells from the neighboring epidermal and mesophyll cells, and so both the osmotic potential and water potential of the guard cells are lowered. These create a water potential gradient between the guard cells and the neighboring cells, making the water move into the guard cells, and resulting in the enlargement of the guard cells that eventually bow outwards causing the stomatal pore to open. Water is supplied from the root through the xylem vessels. Since the xylem vessels are connected to each leaf, the amount of water which the xylem vessels can supply simultaneously would be limited. The number of leaves that lose water would be higher in WIP than in IIL. In WIP, the difference in water potential could contribute to an increase in the amount of water supply itself, but it is unlikely that the same amount of water that flows into an IIL can flow into each leaf of a WIP. As a result, it is expected that WIP would lose more water than IIL. Assuming that the leaf water content of a WIP decreases during photosynthetic induction, it is expected that the turgor pressure in the neighboring epidermal and mesophyll cells would also decrease, resulting in the stomata opening more smoothly with less tension. Why was stomatal opening promoted in two *abcg* mutants with the WIP and IIL? There could be two possibilities: (1) ABA positively closed the stomata in the dark in WT but not in the two *abcg* mutants, (2) the leaf water content was lower because of the smaller amount of ABA in the guard cell resulting in the stomata open more smoothly. More studies are needed to clarify the specific mechanism that promoted stomatal opening more in a WIP than in an IIL.

### ABA-Mediated Prompt Stomatal Response Improves Plant Biomass

Since photosynthesis is the basis for plant growth and yield (Yamori et al., 2016b), researchers have been trying to enhance photosynthetic performance to improve plant biomass and/or yield (Yamori et al., 2016a). In most studies, the target has been to improve leaf photosynthesis under constant conditions (Wang et al., 2014; Simkin et al., 2017). However, in natural environments, various environmental factors, especially light, change dynamically over time (Pearcy et al., 1990; Pearcy, 1990; Yamori, 2016). Therefore, we should explore strategies for optimizing photosynthesis and plant growth in natural environments. Recent work has shown that stomatal conductance, at the onset of a sudden light increase, plays a major role in photosynthetic induction from an analysis of *aba*2-1 mutant in *A. thaliana* (Kaiser et al., 2016). Additionally, it was recently reported that acceleration of stomatal opening and closing caused by introduction of synthetic, blue light-gated K^+^ channel to guard cells enhanced plant growth in *A. thaliana* in the fluctuating light conditions (Papanatsiou et al., 2019).

The present study showed that the photosynthetic induction time at 400 μmol mol^−1^ CO_2_ was shortened in *abcg25* and *abcg40* with a rapid increase in *g*_s_ (**Figure 5**), whereas at a high CO_2_ concentration of 1,500 μmol mol^−1^, the photosynthetic induction time was shortened under both conditions, and the differences in the photosynthetic induction time were eliminated (**Figure 6** and **Table 2**). These results clearly showed that the shortening of the photosynthetic induction time was due to the high *C*_i_ levels caused by the quick stomatal opening upon irradiation. Since the amount of ABA transported to a guard cell was estimated to be lower in *abcg25* and *abcg40* than in WT, the lower ABA concentration consequently promoted stomatal opening upon irradiation, leading to improved photosynthetic induction. Moreover, the plant growth under fluctuating light in the two *abcg* mutants was greater than in WT, indicating that rapid induction improved the efficiency of the total photosynthesis during plant growth. As it has been reported that there are several transporters for ABA (Kuromori et al., 2018), we concluded that an improvement in the stomatal response caused by a slight impairment of the ABA transport could promote a photosynthetic response to a repeated fluctuating light and thus improve the plant biomass under long-term fluctuating light conditions with well-controlled relative humidity. We propose that a consideration of stomatal conductance will be promising approach in terms of improving photosynthesis in natural environments where irradiance always fluctuates.

## Data Availability Statement

All datasets generated for this study are included in the article/supplementary material.

## Author Contributions

All authors conceived and designed the experiments. SS and WY performed the experiments. SS analysed the data and prepared figures and graphs. SS and WY prepared the manuscript, and all the members contributed extensively to its finalization.

## Funding

This study was partly supported by Japan Society for the Promotion of Science (JSPS) [KAKENHI Grant Number: 16H06552, 18H02185, and 18KK0170 (to WY)] and by Ichimura foundation for new technology to WY.

## Conflict of Interest

The authors declare that the research was conducted in the absence of any commercial or financial relationships that could be construed as a potential conflict of interest.
