# Supplementary material for: Whole Irradiated Plant Leaves Showed Faster Photosynthetic Induction Than Individually Irradiated Leaves via Improved Stomatal Opening
Source: Front Plant Sci. 2019 Nov 28;10:1512. doi: 10.3389/fpls.2019.01512 (PMC6892984; doi:10.3389/fpls.2019.01512)
Supplement: Supplementary file 1 [file DataSheet_1.pdf]

Supplemental Data

Whole irradiated plant leaves showed faster photosynthetic induction than individually irradiated leaves via improved stomatal opening

Shunji Shimadzu[1], Mitsunori Seo[2], Ichiro Terashima[1], Wataru Yamori [1,3]*

[1] Department of Biological Sciences, Graduate School of Science, The University of Tokyo, Tokyo, Japan

[2] RIKEN Center for Sustainable Resource Science, Yokohama, Japan

[3] Institute for Sustainable Agro-Ecosystem Services, The University of Tokyo, Nishitokyo, Japan

*** Correspondence:**
Wataru Yamori
wataru.yamori@isas.a.u-tokyo.ac.jp

Figure S1

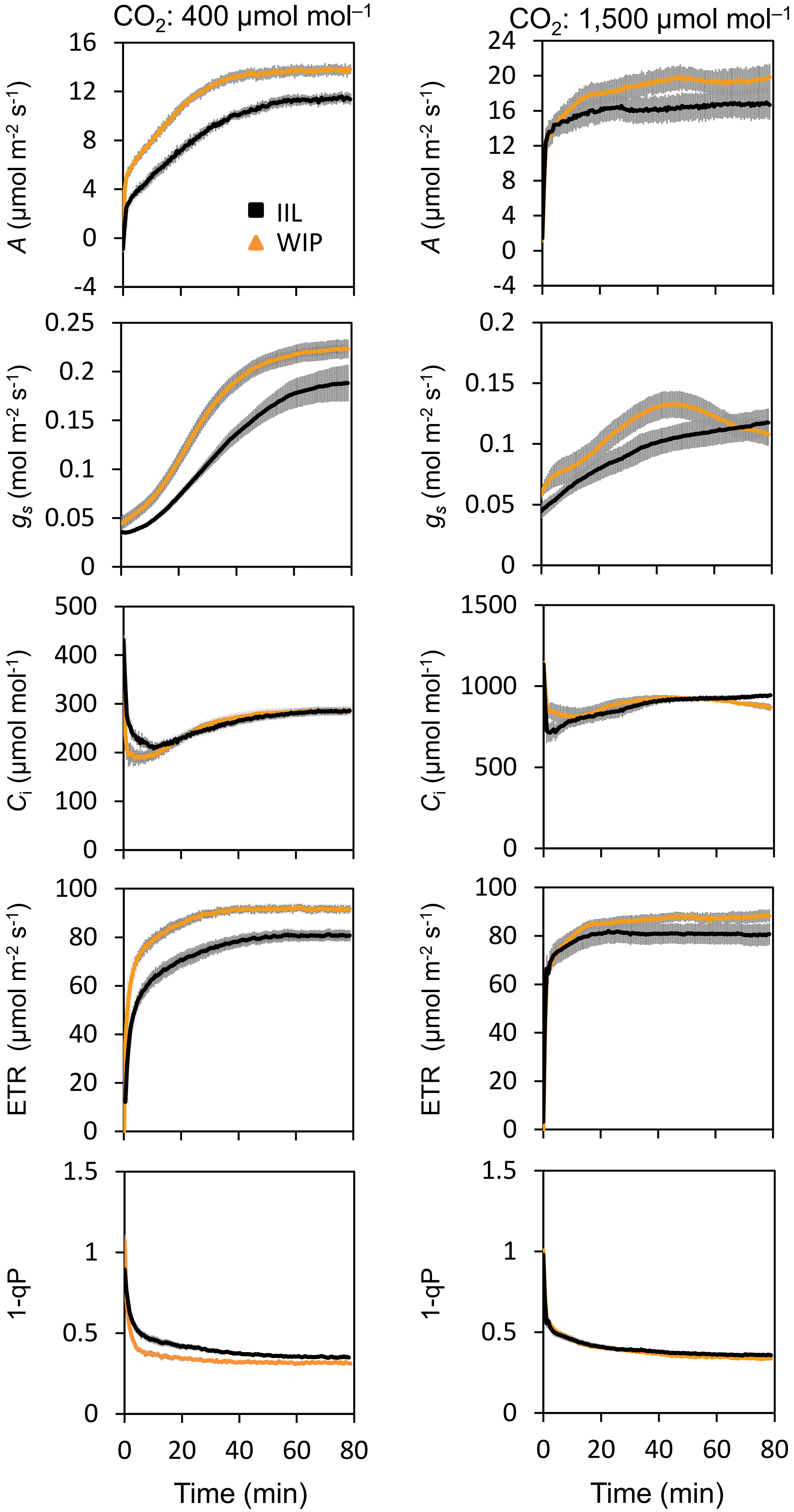

CO2: 400 μmol mol−1
CO2: 1,500 μmol mol−1
A (μmol m-2 s-1)
IIL
WIP
gs (mol m-2 s-1)
Ci (μmol mol-1)
ETR (μmol m-2 s-1)
1-qP
Time (min)

Figure S1

Photosynthetic induction in a leaf of a whole irradiated plant (WIP) and an individually irradiated leaf (IIL) of Arabidopsis WT. CO_2 assimilation rate (A), stomatal conductance (g_s), intercellular CO_2 concentration (C_i), photosynthetic electron transport rate (ETR) and the redox state of the plastoquinone pool (1–qP) were simultaneously measured in an IIL or WIP, at CO_2 concentrations of 400 µmol mol^{-1} and 1500 µmol mol^{-1}. The leaves of plants kept in the dark overnight were used for the experiments. The photosynthetic parameters were recorded every 30 seconds at an irradiance of 500 µmol photons m^{-2} s^{-1} until 80 min. Relative values are shown in Fig. 3. Each data point represents the mean ± standard error for measurements obtained with separate leaves ($n \geq 4$).

Figure S2

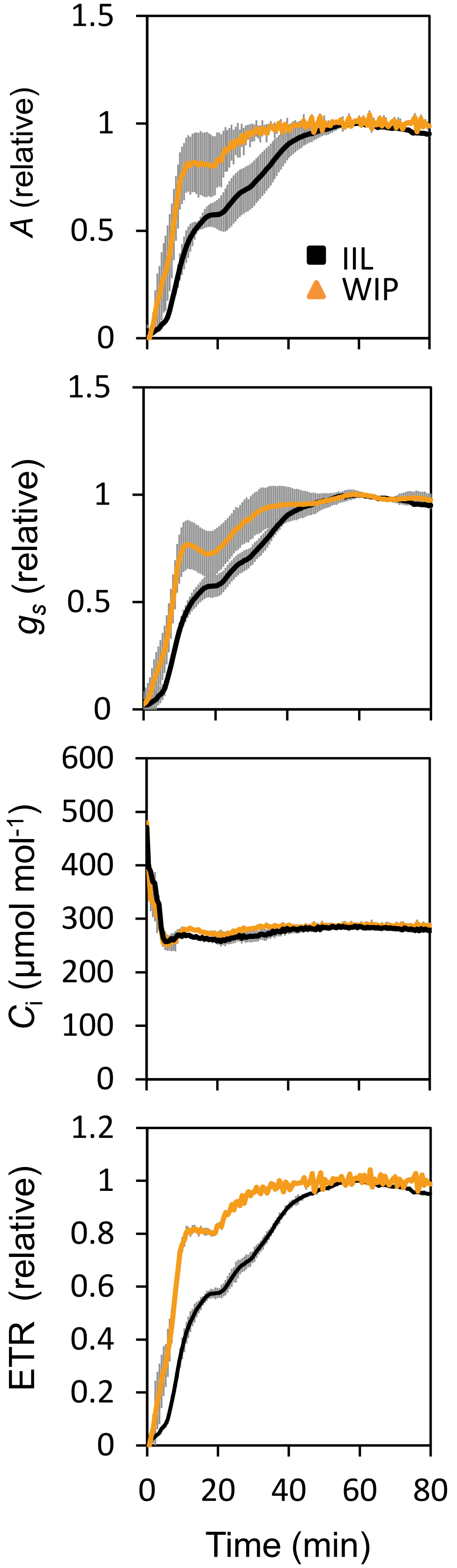

Figure S2

Photosynthetic induction in a leaf of a whole irradiated plant (WIP) and an individually irradiated leaf (IIL) in rice WT. CO_2 assimilation rate (A), stomatal conductance (g_s), intercellular CO_2 concentration (C_i) and the electron transport rate (ETR) were simultaneously measured in IIL or WIP, at CO_2 concentrations of 400 µmol mol^{-1} and 1500 µmol mol^{-1}. Except for C_i, the relative values are shown. The leaves of plants kept in the dark overnight were used for the experiments. The photosynthetic parameters were recorded every 30 seconds at an irradiance of 1000 µmol photons m^{-2} s^{-1} until 80 min. Each data point represents the mean ± standard error for measurements obtained with separate leaves ($n \geq 4$).

Figure S3

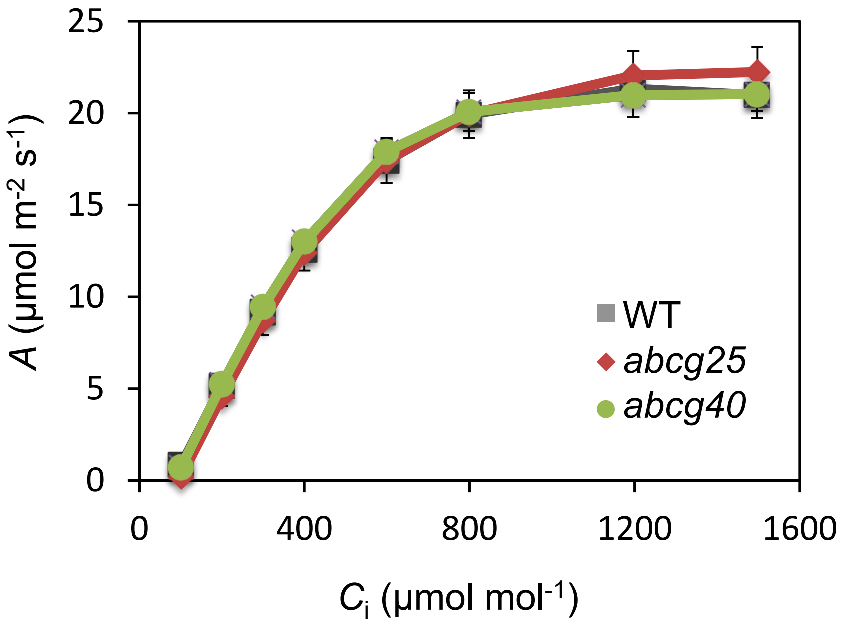

Figure S3
A-C_i curves measured at 500 µmol m^{-2} s^{-1} light. The values represent the mean ± standard error for measurements obtained with separate leaves (n = 4).
